# Cryoballoon vs. pulsed-field ablation for pulmonary vein isolation and roof line ablation in persistent atrial fibrillation with left atrial dilatation

**DOI:** 10.1093/europace/euag058

**Published:** 2026-03-23

**Authors:** Joerg Yogarajah, Patrick Kahle, Julie Hutter, Mirlinda Lüsebrink, Marko Tomic, Andreas Hain, Samuel Sossalla, Thomas Neumann, Malte Kuniss

**Affiliations:** Department of Cardiology, Kerckhoff Heart Center, Campus Kerckhoff, Justus Liebig University Giessen, Beneke Street 2-8, Bad Nauheim 61231, Germany; Department of Cardiology, Kerckhoff Heart Center, Campus Kerckhoff, Justus Liebig University Giessen, Beneke Street 2-8, Bad Nauheim 61231, Germany; Department of Cardiology, Kerckhoff Heart Center, Campus Kerckhoff, Justus Liebig University Giessen, Beneke Street 2-8, Bad Nauheim 61231, Germany; Department of Cardiology, Kerckhoff Heart Center, Campus Kerckhoff, Justus Liebig University Giessen, Beneke Street 2-8, Bad Nauheim 61231, Germany; Department of Cardiology, Kerckhoff Heart Center, Campus Kerckhoff, Justus Liebig University Giessen, Beneke Street 2-8, Bad Nauheim 61231, Germany; Department of Cardiology, Kerckhoff Heart Center, Campus Kerckhoff, Justus Liebig University Giessen, Beneke Street 2-8, Bad Nauheim 61231, Germany; Department of Cardiology, Kerckhoff Heart Center, Campus Kerckhoff, Justus Liebig University Giessen, Beneke Street 2-8, Bad Nauheim 61231, Germany; Department of Cardiology, Medical Clinic I, Justus Liebig University Giessen, Giessen 35392, Germany; Cardio-Pulmonary Institute (CPI), Excellence Cluster, Justus-Liebig-University Giessen, Germany; Department of Cardiology, Kerckhoff Heart Center, Campus Kerckhoff, Justus Liebig University Giessen, Beneke Street 2-8, Bad Nauheim 61231, Germany; Department of Cardiology, Kerckhoff Heart Center, Campus Kerckhoff, Justus Liebig University Giessen, Beneke Street 2-8, Bad Nauheim 61231, Germany

Single-shot pulsed-field ablation (PFA) systems have recently emerged as promising alternatives to cryoballoon (CB) ablation.^1^ However, comparative long-term data on ablation beyond pulmonary vein isolation (PVI) with these systems remain limited. Additional left atrial roof line ablation (LARA) using either CB or single-shot PFA in patients with persistent atrial fibrillation (AF) and left atrial (LA) enlargement may be an effective strategy.^2,3^

The aim of this observational study was to perform a head-to-head comparison of CB and PFA in patients with persistent AF and dilated atria undergoing first-time AF ablation, combining fluoroscopy-guided PVI and LARA.

We analyzed consecutive patients at Kerckhoff Heart Centre undergoing first-time fluoroscopy-guided PVI with adjunctive LARA using either a 28-mm CB ballon (Arctic Front Advance Pro™, Medtronic) or pentaspline or circular PFA catheter (FARAPULSE™ system, Boston Scientific, Marlborough, MA; PulseSelect™ system, Medtronic, Minneapolis, MN) in patients with persistent AF and left atrial enlargement (LA area >20 cm^2^) between November 2023 and October 2024. Antiarrhythmic drugs were discontinued after the 90-day blanking period unless early recurrence occurred. The primary endpoint was 12-month freedom from atrial arrhythmia (AF/AT >30 s) following a 90-day blanking period. Follow-up included clinic visits with 12-lead ECGs and 24 h Holter monitoring at 3, 6, and 12 months. The study was approved by the Ethics Committee of Justus Liebig University Giessen (AZ 83/24) and performed following the principles of the Declaration of Helsinki.

Procedures were performed under conscious sedation for CB (diazepam/piritramide) and deep sedation for PFA (propofol/remifentanil; initial cases under general anaesthesia). Phrenic nerve function was monitored during CB ablation, whereas for PFA it was assessed by capture testing or post-ablation monitoring.^3^

Adjunctive LARA after PVI was performed using overlapping CB freezes or sequential PFA applications along the LA roof from the LSPV to the RSPV, with lesion assessment by differential pacing as previously described.^2,3^ Assessments were performed as part of the standard workflow, without predefined waiting periods or observation intervals.

In this study, the primary comparison was performed between CB and PFA catheters. In addition, an exploratory subgroup analysis was conducted within the PFA cohort, separating pentaspline and circular systems.

A total of 125 consecutive patients were included [CB 65; PFA 60 (pentaspline 30; circular 30)] with balanced baseline characteristics between the groups (*Figure 1A*).

**Figure 1:**
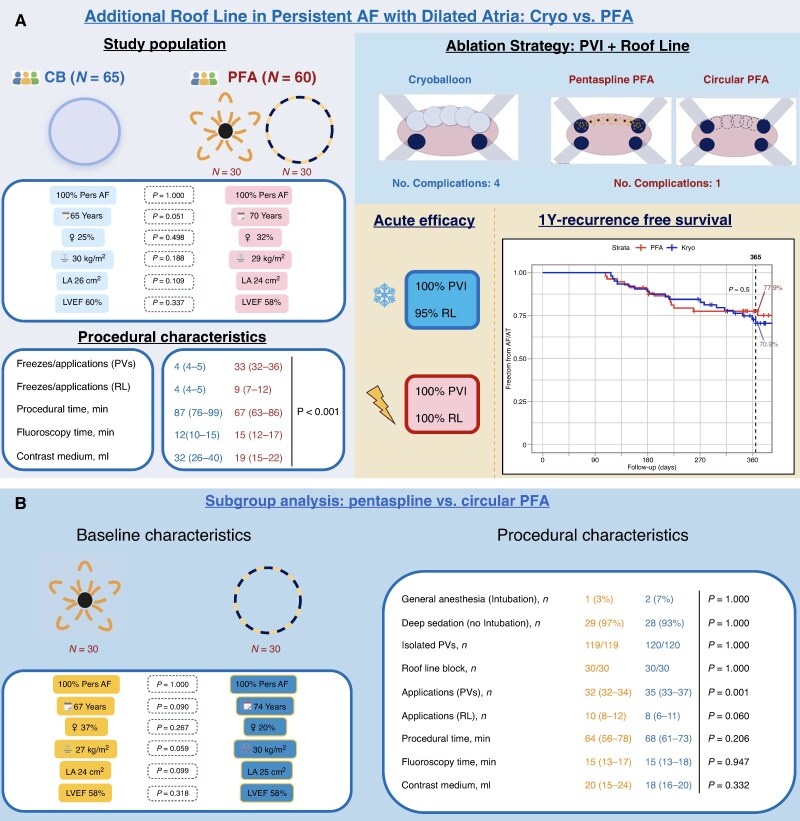
(*A*) Main analysis between CB and PFA catheters for combined PVI and RL ablation. (*B*) Subgroup analysis between pentaspline and circular PFA systems for PVI and RL. AF, atrial fibrillation; CB, cryoballoon; LA, left atrial area; LVEF, left ventricular ejection fraction; PFA, pulsed field ablation; PV, pulmonary vein; PVI, pulmonary vein isolation; RL, roof line

CB procedures showed longer total procedure and LA dwell times than PFA, but shorter fluoroscopy time. Acute PVI was achieved in 100% of both groups. CB required fewer roof line applications/freezes than PFA catheters with conduction block achieved in 95% and 100%, respectively (*P* = 0.11). Contrast medium use was higher in CB procedures. Overall complication rates were low and comparable between CB and PFA (4 vs. 1; *P* = 0.37). In the CB group, one sustained phrenic nerve palsy occurred but resolved within 1 year; the remaining complications were minor vascular events not requiring intervention. The single PFA-related complication was a minor vascular event in the circular PFA group; none occurred in the pentaspline group.

Within the PFA cohort, baseline and procedural characteristics were largely similar between pentaspline and circular systems, except for a higher number of PVI applications in the circular PFA group (*Figure 1B*).

Median follow-up was 393 days (IQR 348–504), with no significant difference between CB and PFA (*P* = 0.298). Kaplan-Meier-estimated 1-year arrhythmia-free survival was 70.9% for CB and 77.9% for PFA (log-rank *P* = 0.50; *Figure 1A*), with no significant differences among the three ablation systems (CB 70.9%, pentaspline 79.5%, circular 76.7%; *P* = 0.55). During follow-up, among all arrhythmia recurrences, atrial tachycardia (AT) or atrial flutter (AFL) occurred in 27% (6/22), 25% (2/8), and 8% (1/12) of patients in the CB, pentaspline, and circular PFA groups, respectively; most recurrences were AF. Reablation was performed in 5/65 (7.7%), 4/30 (13.3%), and 2/30 (6.7%) patients in the CB, pentaspline, and circular PFA groups, respectively, with the roof line still blocked in 3/5, 2/4, and 1/2 cases. In patients with an unblocked roof line, roof-dependent AT occurred in 3 of 6 cases.

This study is the first to directly compare LARA using CB and single-shot PFA systems during fluoroscopy-guided PVI in patients with persistent AF and dilated atria. Both technologies achieved high acute success and similar 1-year arrhythmia-free survival while maintaining favourable safety profiles. Procedural characteristics differed between systems, reflecting device-specific differences, but overall outcomes were largely comparable.

The PFA cohort included pentaspline and circular systems with different electrode geometries and energy delivery patterns.^4^ However, both achieved high acute roof line block and PVI rates, with only minor differences in the number of applications. Sequential applications with single-shot PFA catheters may provide broader tissue coverage than CB, thereby facilitating effective roof line formation. The pentaspline system may allow extension to posterior wall or box isolation.^5^

Procedural workflow could be optimized using 3D navigation. Moreover, sedation strategies may influence practical implementation, as CB can be performed under lighter sedation.

So far, most data on ablation beyond PVI refer to radiofrequency ablation, with controversial long-term results.^6–8^ However, patients with dilated atria and persistent AF remain underrepresented. Long-term data on ablation beyond PVI with PFA or CB mainly concern posterior wall isolation, showing favourable outcomes.^5,9^ Comparative evidence for LARA, particularly with PFA in enlarged atria, remains scarce. LARA may reduce arrhythmogenic substrate in persistent AF and prevent roof-dependent atrial arrhythmias when durable conduction block is achieved,^10^ whereas incomplete lines may promote arrhythmia.

This study is limited by its single-centre, observational design, modest sample size, and reliance on intermittent ECG/Holter follow-up, which may underestimate asymptomatic recurrences.

In conclusion, our findings firstly support the use of adjunctive LARA with CB and PFA single-shot devices in challenging persistent AF patients with LA dilatation, demonstrating favourable feasibility, safety, and efficacy, and potentially offering better rhythm outcomes than PVI-only approaches. However, these results are hypothesis-generating, given the observational design, mixed use of single-shot technologies, and absence of a PVI-only control group, which precludes direct comparisons. Future multicenter, randomized studies are warranted to evaluate long-term lesion durability and clinical benefit, including direct comparison with PVI-only strategies.

## Data Availability

The data presented in this study are available on request from the corresponding authors.
